# Assessment of the Readability and Quality of Online Patient Education Material for Chronic Medical Conditions

**DOI:** 10.3390/healthcare10020234

**Published:** 2022-01-26

**Authors:** Peter Minh Hoang, Courtney van Ballegooie

**Affiliations:** 1 Department of Internal Medicine, Cumming School of Medicine, Calgary, AB T2N 4N1, Canada; 2 Experimental Therapeutics, BC Cancer Research Institute, Vancouver, BC V5Z 1L3, Canada; cballegooie@bccrc.ca; 3 Faculty of Medicine, University of British Columbia, Vancouver, BC V6T 1Z3, Canada

**Keywords:** geriatrics, health literacy, information literacy, health services for the aged, health education, consumer health information, patient care, health information exchange

## Abstract

Patient education materials (PEMs) were assessed from chronic health condition associations to determine their quality and if they were above the 6th grade reading level (GRL) recommended by the Centers for Disease Control and National Institutes of Health. PEMs from 55 associations were assessed for their GRL using ten readability scales and underwent a difficult word analysis. The associations had their quality assessed using two methods: the Journal of the American Medical Association (JAMA) Benchmarks and Health on the Net Foundation Code of Conduct certification (HONCode). Two thousand five hundred and ninety PEMs, collected between June and November 2021, were analyzed. The overall GRL average was 10.8 ± 2.8, with a range of 0 to 19. Difficult word analysis showed that 15.8% ± 4.8 contained complex words of 3 or more syllables and 25.7% ± 6.3 contained words which were unfamiliar. No association displayed all four indicators of quality according to JAMA Benchmarks or held an up-to-date HONCode certification. The PEM readability continues to be written at a level above the recommended GRL. Additionally, the lack of quality indicators from the associations’ websites may make it difficult for older adults to identify the sources as credible. This represents an opportunity to optimize materials that would be understood by a wider audience.

## 1. Introduction

Older adults are the most likely to experience chronic health conditions, which are the leading causes of morbidity and mortality. This population has also been shown to have lower levels of health literacy relative to the general population [1,2,3]. Health literacy is defined as an individual’s ability to access, understand, and utilize information to create an informed decision regarding their health, and is correlated to reading level [3,4,5,6]. Older adults who have higher levels of health literacy have been shown to have better management of their medical conditions, including diabetic and hypertension targets, medication adherence, and healthy lifestyle behaviors [7,8,9,10]. While the Centers for Disease Control (CDC) already recommend that health information provided to patients be written two levels below the population’s average grade reading level (GRL), corresponding to a 6th GRL, an even lower GRL may be required in those over the age of 65 [11].

Online health information has provided a unique opportunity to improve health literacy. Population studies have shown that 67% of older adults use the Internet, and up to 50% use the Internet for health information [12,13,14]. Accordingly, online patient education materials (PEMs) provide an opportunity to further improve health education. Previous studies have shown that PEMs are written at a grade level above what most American adults would be able to understand [15,16,17,18,19,20,21]. Similarly, two studies assessing online patient health information from geriatric associations identified that their average grade reading level was above the recommended 6th GRL [22,23]. Our study aims to examine PEMs from 55 associations that provide information on specific chronic health conditions common to older adults, based on 10 validated readability scales with disease cluster specific recommendations [24]. In addition, the quality of the associations was assessed in order to determine if the associations’ websites would easily allow one to identify them as a credible source of health-related information.

## 2. Materials and Methods

### 2.1. Sample Collection

From June 2021 to November 2021, all internet-based PEMs were extracted from the associations’ websites and pooled into their respective disease cluster (Table 1). The specific associations identified are described in Supplementary Table S1 and included both national organizations and foundations.

The most common conditions experienced by older adults were selected from CDC data, such as cardiovascular disease (including stroke), chronic lung disease (including asthma, chronic obstructive pulmonary disease, and sleep apnea), arthritis, chronic kidney disease, dementia, and osteoporosis [2,25]. Risk factors associated with chronic kidney disease and heart disease, such as hypertension and diabetes, were also included if there were PEMs from the respective associations identified. Visual deficits and urinary incontinence were also identified as common conditions older adults experience, and thus were included in the analysis [26,27]. The downloaded PEMs included materials describing topics related to the specific disease clusters with an intended use by patients and their caregivers. Materials were excluded if they had an intended use by health care providers. Only the top five most common cancers, as identified by the American Cancer Society by gender, were selected for assessment. For men, this included prostate, lung, colorectal and bladder cancer as well as melanoma, while for females this included breast, lung, colorectal, uterine, and thyroid cancers [28]. Given that the majority of cancers are diagnosed over the age of 55, this study aimed to capture what older adults are most likely to experience [28]. Additionally, it should be noted that certain associations contain PEMs that span multiple medical conditions, particularly the American Geriatric Society and National Institute on Aging. If a document was in Portable Document Format (PDF), it was manually converted to a Microsoft Word document (Microsoft Corp) for further analysis. Text sections of nonmedical information such as diagrams, tables, page numbers, disclaimers, and webpage navigation were excluded from assessment [15,18]. 

### 2.2. Document Readability Analysis

A readability assessment was then performed on the PEMs using the software package Readability Studio professional edition version 2019.3 (Oleander Software, Ltd., Pune, India). The readability grade level scales included the Coleman–Liau Index (CLI), New Dale–Chall (NDC), Degrees of Reading Power and Grade Equivalent test (DRP-GE), Flesch–Kincaid Grade Level (FK), Gunning Fog Index (GF), New Fog Count (NFC), Simple Measure of Gobbledygook Index (SMOG), and Ford, Caylor, Sticht (FORCAST) scale. Two graphical scales were included, the Raygor Readability Estimate Graph (RREG) and the Fry Readability Graph (FRG). These ten scales provide an estimate of the GRL requirement and are frequently used when assessing medical text, and they offer externally validated measures of readability [15,18]. Given the formulaic variation for the weighting and parameters used in these readability scales, an assessment of PEMs using multiple formulas provides greater assurance in the calculated GRL.

**Figure 1:**
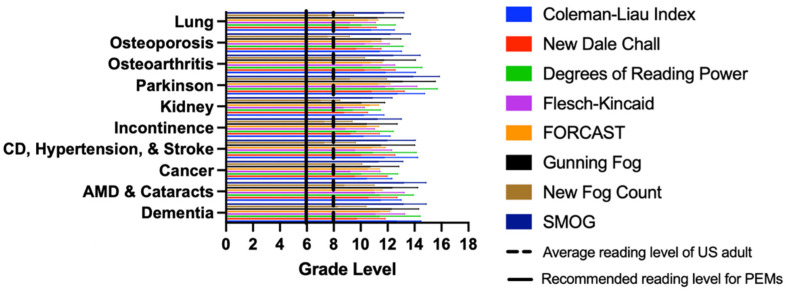
Mean grade level of online patient education materials found for each disease cluster (Lung disease, Osteoporosis, Osteoarthritis, Parkinson’s, Kidney disease, Incontinence, Cardiovascular disease [CD], Hypertension, and Stroke, Cancer, Age related macular degeneration [AMD] and Cataracts, and Dementia) using eight numerical scales, including the Coleman–Liau Index, New Dale–Chall, Degrees of Reading Power and Grade Equivalent test, Flesch–Kincaid Grade Level, Gunning Fog Index, New Fog Count, Simple Measure of Gobbledygook Index (SMOG), and Ford, Caylor, Sticht (FORCAST) scale.

Once the PEMs were extracted from the 55 associations, PEMs were then formatted to account for non-narrative text. This included the alteration of bullet points. PEMs were individually edited to create high- and low-sentence documents wherein bullet points were treated as individual sentences or a single sentence, respectively, which were then subsequently averaged for numerical indices [21,22]. Their readability level using the eight numerical scales can be seen in Figure 1, generated using Prism 9 software, and the two graphical scales can be seen in Supplementary Figures S1 and S2, generated using Readability Studio.

### 2.3. Difficult Word Analysis

Individual words from each of the PEMs were extracted using Readability Studio. The analysis included the identification of the percentage of complex words (3+ syllable words), the percentage of long words (6+ character words), as well as the percentage of unfamiliar words according to the NDC criteria. Words were compared to the NDC word list as well as the New General Service List (NGSL), and those that appeared in either of the lists were removed and considered as non-jargon words. All words with 3 syllables or more were then extracted. Hyphenated words were only included if one or more of the components contained unfamiliar words. Three-syllable words, prioritized by their frequency for each of the disease clusters and the number of PEMs they appeared in, were then combined with their alternate tenses. Alternative words were then proposed for the most frequent 3-syllable words using the Readability Studio Software, the Merriam-Webster Thesaurus, or a medical doctor (P.M.H.), in order to identify synonyms that could decrease the difficulty of the word by an individual word’s syllables and/or character length. Alternatives also provided short phrases that either avoided medical jargon or were in vernacular language for certain difficult words [29].

### 2.4. Quality Analysis

A quality analysis was performed using two well-established, validated tools including the Health On the Net (HON) Foundation Code of Conduct (HONCode) and Journal of the American Medical Association (JAMA) benchmarks. HONCode evaluates the credibility and reliability of information for medical and health websites using the following criteria: disclosure of authors’ qualifications, attribution/citation of sources, data protection, justifiability, transparency, and disclosure of sources of funding and advertising [30,31,32]. Associations incur a fee in order to be evaluated by HONCode and receive a certification if they meet HONCode criteria. Each association, regardless of whether it authored PEMs for the disease clusters of interest, had their HONCode certification status verified. The JAMA benchmarks were also used to assess the quality of each website. This instrument evaluates the presence of 4 components: authorship (including affiliations and credentials), references, disclosure (including ownership, advertising policy, sponsorship, and conflicts of interests), and currency (e.g., date of creation/update) [31,33]. Each of the associations that contained PEMs relevant to this study were further evaluated. This included extracting five PEMs from each of the associations and evaluating them using two independent reviewers according to the JAMA benchmark criteria. The associations then had the mode taken for each of the criteria of the five PEMs to achieve their final JAMA benchmark score.

### 2.5. Statistical Methods

The graphical data in Figure 1 was reported as the arithmetic mean of each numerical scale. The average of the eight numerical readability tests for each of the disease types underwent a one-way Analysis of Variance (ANOVA) with a Tukey’s test comparing the disease types [21,22]. A pooled standard deviation was used to calculate the standard deviation whenever disease types were combined, such as for the difficult word analysis, in order to ensure that the number of PEMs extracted for each disease cluster was taken into consideration. Statistics were analyzed using Graph Pad Prism 9.

## 3. Results

### 3.1. Document Readability Analysis

A total of 2590 PEMs were downloaded and assessed from 55 associations for ten chronic medical condition clusters. The average grade reading level of the eight readability scales are as follows: age-related macular degeneration and cataracts (11.2 ± 1.6), cancer (10.0 ± 1.8), dementia (11.3 ± 1.6), cardiovascular disease, heart attack, and stroke (10.1 ± 1.9), kidney-associated diseases (9.5 ± 1.3), osteoarthritis (10.8 ± 2.0), osteoporosis (10.6 ± 1.7), Parkinson’s disease (11.9 ± 1.8), urinary incontinence (9.7 ± 1.8), and lung-associated diseases (10.0 ± 2.2). There was a significant difference identified in the ANOVA (*p* < 0.0001). Parkinson’s was identified as the most difficult to read relative to all other diseases. The overall mean for all medical condition clusters was 10.8 ± 2.8, with a grade level range of 0 to 19; zero suggests that the PEM can be read by any person. The numerical indicators showed that 95.3% and 82.7% of PEMs were above the 6th and 8th GRL, respectively. Figure 1 illustrates a summary of the results for the various disease clusters, showing each of the eight readability scales used. The RREG of the high sentence estimate (Supplementary Figure S2) ranges from a 3rd grade reading level to a grade level equivalent to that of a professor level (grade 17), with 98.5% and 89.5% exhibiting a grade level above six and eight, respectively. The FRG of the high sentence estimate, as seen in Supplementary Figure S1, ranges from a 3rd grade to a 17th grade (university) reading level, with 99.0% and 89.6% exhibiting a grade level above six and eight, respectively.

### 3.2. Difficult Word Analysis

From the difficult word analysis, it was found that, on average, the PEMs for all disease clusters were comprised of 15.8% ± 4.8% of complex words that contained three or more syllables, 35.2% ± 5.6% of words that contained six or more characters, and 25.7% ± 6.3% of words that were unfamiliar. Supplementary Table S2 describes the top ten most frequent difficult words separated by disease cluster, and Supplementary Table S3 describes the complex, long, and unfamiliar analysis for each of the individual disease clusters. Supplementary Table S4 displays the ANOVA and pairwise comparison of the difficult word analysis, which indicated that Parkinson’s disease was the most difficult in all three difficult word analyses performed (*p* < 0.001).

### 3.3. Quality Analysis

**Figure 2:**
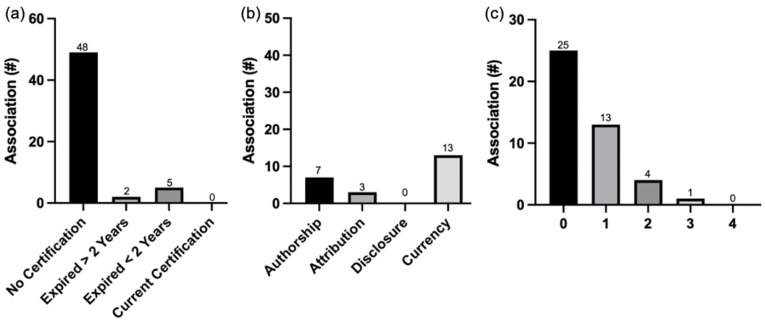
The quality analyses using the Health On the Net (HON) Foundation Code of Conduct (HONCode) and JAMA benchmarks. (**a**) The number of associations that either have never had a HONCode certification, have a certification that expired over two years ago, have a certification that expired under two years ago, or have a current certification. (**b**) The number of associations, out of 43 associations, that display each of the individual JAMA benchmarks, including authorship, attribution, disclosure, and currency. (**c**) The number off associations that display zero, one, two, three, or all four of the JAMA benchmarks.

From the quality analysis, it was found that none of the associations held a currently valid HONCode certification (Figure 2a). Only seven of the 55 associations ever held a certification at any point in time. Additionally, the majority of the associations (58%) displayed zero JAMA Benchmark quality indicators (Figure 2c). Authorship and currency were identified as the most commonly reported quality indicators and disclosure as the least common indicator (Figure 2b).

## 4. Discussion

### 4.1. Implications

Online patient health materials are a potential mechanism to improve the management of chronic diseases, as they have been shown to promote non-pharmacological healthy lifestyle behaviors and improve adherence to physician advice [34,35]. Therefore, they should be written at a level consistent with the health literacy of the targeted population and be easily identifiable as credible sources of health information. Our study has identified that PEMs from associations for chronic medical conditions are written at a GRL of 10.8 ± 2.8. In addition, the graphical scales identified that over 98% of PEMs were written above the recommended 6th grade level. As chronic medical conditions are common in older adults, this suggests that PEMs from these associations are not written at the 6th GRL that is recommended as being understood by most American older adults.

This study aimed to identify whether different medical topics common to older adults may have different levels of readability, which may help identify PEMs that require more focused efforts to improve readability. Grouped by disease cluster, the medical conditions that had the lowest readability average were kidney disease, cancer, and urinary incontinence. Certain associations have begun to use readability indices in the development of their PEMs; for example, the National Cancer Institute uses the SMOG readability test as its gold standard [36]. In addition, certain disease clusters only had a small number of associations creating PEMs, which may confound readability either positively or negatively depending on whether readability was factored in during their authorship. Conditions in which there is typically a higher frequency of medical jargon due to the disease, pathophysiology, and its treatment (e.g., dementias, Parkinson’s disease, and macular degeneration), may be more likely to have higher grade level requirements. The difficult word analysis identified the top ten most frequently used terms that were complex, either by syllabic count, character count, or unfamiliarity. The majority of these words were medical jargon. Overall, to improve the readability of the PEMs, associations should not only consider their word choices, such as choosing short, familiar, and mono- or bi-syllabic words, but also the PEMs’ format (e.g., a simple style and layout) and use of multimedia (e.g., visuals and illustrations), as both have been shown to assist in comprehension [11,37,38,39]. Associations should give additional consideration to the disease clusters that were identified as being the most difficult to read (e.g., Dementia and Parkinson’s).

Several other studies have examined PEM readability across many surgical and medical specialties outside of geriatrics [16,17,18,19,36,40,41,42,43,44]. In addition, we have previously assessed PEMs from geriatric-specific associations. Overall, these studies have shown that the GRL of PEMs remain above the GRL of the average American [22]. Compared to PEMs of internal medicine diagnoses and its subspecialties, we found a similar trend wherein PEMs were, on average, above the recommended 6th GRL [15,45,46]. Compared to studies examining geriatric associations, the overall GRL of these chronic disease associations was similar to previous studies, suggesting that even if older adults use sources outside of geriatric specific organizations, they will likely still face difficulties in finding accessible and reliable information for their health concerns [22,23]. In addition to readability, this paper is the first to identify that none of the associations related to chronic diseases held an up-to-date HONcode certification, a quality indicator gold standard, nor did the majority of the associations display any JAMA Benchmark quality indicators (Figure 2). The most (authorship and currency) and least (discloser) frequently identified quality indicator, as identified in this study, aligns well with previous findings in other fields of medicine [33]. Quality has been assessed in many different medical fields in the U.S., such as surgery, oncology, ophthalmology, and infectious disease, and they have come to similar conclusions, citing the need for higher quality information [32,33,47]. This study has identified a need for associations related to chronic diseases to better display quality indicators in order for patients and caregivers to navigate medical information on the internet and identify credible sources of health information.

### 4.2. Limitations

This study has several limitations. Certain chronic medical conditions may be understood by many older adults, such as arthritis, Parkinson’s disease, or Alzheimer’s dementia, but would have a high GRL as many of the readability indices use syllabic count, which can potentially overestimate GRL. That being said, medical jargon that has fewer syllables, such as renal or Merkel cell cancer, may not be accurately captured within readability scales. Many of the PEMs also explain the medical condition that is being described, but repetition of medical jargon is frequently necessary, which may increase the overall GRL. Due to the limitations of the text-based readability indices, visual representation components from PEMs could not be analyzed. It is well known that visual aids can effectively and efficiently convey medical information and should be considered in future studies. Additionally, the comprehension of health information depends on many parameters, including health and cultural experiences as well as the specific goals when reading PEMs, which were not evaluated in this study [37]. Authors of PEMs can consider the various following guidelines in the development of their materials, including the NIH Clear & Simple and the United States General Services Administration University Design and Accessibility guides [38,39].

## 5. Conclusions

Over 2500 PEMs from 55 associations relating to specific medical conditions that older adults commonly experience have an average GRL of 10.8 ± 2.8, which is significantly above the recommended 6th GRL. Our study identifies an opportunity to provide highly accessible and accurate medical information for patients, but it is imperative that these be written at a level that can be understood and easily identified as credible sources of information by the majority of older adults. Associations should consider the use of readability scales in their PEM design and must better display the quality of their information, either through credible certification or the incorporation of quality benchmarks. Future studies incorporating patient understanding can be considered to assess education material readability among a representative sample of older adults.

## Figures and Tables

**Table 1:** Number of associations and patient education documents of chronic medical conditions.

Disease Cluster	Associations, No.	Documents, No.
Age-Related Macular Degeneration and Cataracts	11 *	164
Cancer ^a^	11 *	628
Dementia	7 *	243
Cardiovascular Disease, Hypertension and Stroke	5 *	402
Kidney	2	262
Osteoarthritis	8 *	93
Osteoporosis	12 *	66
Parkinson’s	6 *	244
Urinary Incontinence	2	80
Lung	8	408

* Identifies the presence of associations that provided PEMs on medical conditions across more than one disease cluster. ^a^ Only the top five most common cancers, as identified by the American Cancer Society by gender, were selected for assessment, which were prostate, lung, colorectal, urinary bladder cancers as well as melanoma in males, and breast, lung, colorectal, uterine, and thyroid cancer in females.

## Data Availability

Data may be made available upon request.

## Supplementary Materials

The following supporting information can be downloaded at: https://www.mdpi.com/article/10.3390/healthcare10020234/s1, Table S1: Associations identified for each chronic disease cluster; Table S2: Difficult Words with Alternative Word Recommendations; Figure S1: Fry readability graph assessment of all high sentence estimate online patient education materials by disease cluster; Figure S2: Raygor readability estimate graph of all high sentence estimate online patient education materials by disease cluster; Table S3: Difficult words analysis displaying the mean and standard deviation of the % 3+ syllable words, % 6+ character words, and % unfamiliar words found in the patient education material (PEMs) of each of the disease clusters; Table S4: Difficult word analysis statistics: Analysis of variance (ANOVA) and pairwise comparison of the difficult words analyses [e.g., the % 3+ syllable words, % 6+ character words, and % unfamiliar words found in the patient education material (PEMs) of each of the disease clusters].

## Abbreviations

Age Related Macular Degeneration	(AMD)
Analysis of variance	(ANOVA)
Centers for Disease Control	(CDC)
Cardiovascular Disease	(CD)
Coleman-Liau index	(CLI)
Degrees of reading power	(DRP)
Flesch-Kincaid grade level	(FK)
Ford, Caylor, Sticht	(FORCAST)
Fry readability graph	(FRG)
Grade equivalent	(GE)
Gunning Fog index	(GF)
Grade reading level	(GRL)
Health On the Net Foundation Code of Conduct	(HONCode)
Journal of the American Medical Association	(JAMA)
New Dale-Chall	(NDC)
New Fog Count	(NFC)
New general service list	(NGSL)
Patient education material	(PEM)
Portable document format	(PDF)
Raygor readability estimate graph	(RREG)
Simple measure of gobbledygook index	(SMOG)
