# Supplementary material for: Assessment of the Readability and Quality of Online Patient Education Material for Chronic Medical Conditions
Source: Healthcare (Basel). 2022 Jan 26;10(2):234. doi: 10.3390/healthcare10020234 (PMC8872454; doi:10.3390/healthcare10020234)
Supplement: Supplementary file 1 [file healthcare-10-00234-s001.zip › healthcare-1559058-supplementary.pdf]

**Supplementary Information Table of Contents**

**Supplementary Table S1.** Associations identified for each chronic disease cluster.

**Supplementary Table S2.** Difficult words with alternative word recommendations.

**Supplementary Figure S1.** Fry readability graph assessment of all high sentence estimate online patient education materials by disease cluster.

**Supplementary Figure S2.** Raygor readability estimate graph of all high sentence estimate online patient education materials by disease cluster.

**Supplementary Table S3.** Difficult words analysis displaying the mean and standard deviation of the % 3+ syllable words, % 6+ character words, and % unfamiliar words found in the patient education material (PEMs) of each of the disease clusters.

**Supplementary Table S4.** Difficult word analysis statistics: Analysis of variance (ANOVA) and pairwise comparison of the difficult words analyses [e.g. the % 3+ syllable words, % 6+ character words, and % unfamiliar words found in the patient education material (PEMs) of each of the disease clusters].

| **Supplementary Table S1.** Associations identified for each chronic disease cluster. | |
|---|---|
| **Disease** | **Associations** |
| Age Related Macular Degeneration and Cataracts | 1) American Academy of Ophthalmology, 2) American Association of Ophthalmic Oncologists and Pathologists[a], 3) American Society of Cataract and Refractive Surgery[a], 4) American Society of Ophthalmic Plastic and Reconstructive Surgery[a], 5) American Society of Retina Specialists American, 6) Uveitis Society[a], 7) Cornea Society[a], 8) North American Neuro-Ophthalmology Society[a], 9) American Macular Degeneration Foundation, 10) American Geriatric Society, and 11) National Institute on Aging |
| Dementia | 1) Dementia Society of America, 2) Alzheimer's Society, 3) Alzheimer's Association, 4) American Brain Society, 5) American Geriatric Society, 6) National Institute on Aging, and 7) American Academy of Ophthalmology |
| Parkinson's Disease | 1) American Parkinson's Disease Association, 2) American Society of Gene and Cell Therapy, 3) American Brain Society, 4) National Parkinson Foundation, 5) American Geriatric Society, and 6) National Institute on Aging |
| Cancer | 1) American Cancer Society, 2) American Lung Association, 3) American Dermatological Association[a], 4 American Academy of Dermatology, 5) American Geriatric Society, 6) National Institute on Aging, 7) American Breast Cancer Foundation, 8) National Breast Cancer Foundation, 9) National Cancer Institute, 10) National Colorectal Cancer Roundtable, and 11) Prostate Cancer Foundation |
| Cardiovascular Disease, Hypertension, and Stroke | 1) American Heart Association, 2) American Stroke Foundation, 3) American Brain Society, 4) American Geriatric Society, 5) National Institute on Aging |
| Osteoarthritis | 1) Arthritis Foundation, 2) American College of Rheumatology, 3) American Rheumatology Network[a], 4) American Arthritis Society, 5) American Society of Surgery for the Hand 6) National Institute of Arthritis and Musculoskeletal and Skin Disease (NIH), 7) American Geriatric Society, 8) National Institute on Aging |
| Kidney Associated Diseases | 1) Kidney Fund and 2) National Kidney Foundation |
| Urinary Incontinence | 1) National Association for Continence and 2) Urology Care Foundation |
| Osteoporosis | 1) National Osteoporosis Foundation, 2) American Society for Bone and Mineral Research[a], 3) American Association of Clinical Endocrinologists, 4) American Thyroid Association[a], 5) Endocrine Society, 6) American Bone Health, 7) Arthritis Foundation, 8) American Geriatric Society, 9) National Institute on Aging, 10) American College of Rheumatology, 11) National Institutes of Health, 12) Own the Bone The American Orthopaedic Association[a] |
| Lung Associated Diseases | 1) American Lung Association, 2) American Sleep Apnea Association, 3) American Thoracic Society, 4) Asthma and Allergy Foundation, 5) |

| | |
|---|---|
| | Chest Foundation, 6) Myositis Association, 7) Pulmonary Foundation Association, 8) Pulmonary Hypertension Association |

[a]Indicates that there were no patient education materials identified for the indicated disease type at that association

**Supplementary Table S2.** Difficult words with alternative word recommendations.

| Disease Cluster | Difficult Word[a] | Frequency | Alternatives[b] |
|---|---|---|---|
| Age Related Macular Degeneration and Cataracts | | | |
| | (Age Related) Macular Degeneration (AMD) | 1373 | N/A |
| | Retina (-l, -s) | 379 | N/A |
| | Ophthalmology [Ophthalmologist (-s)] | 261 | Eye [Doctor] |
| | Cataract (-s) | 595 | Clouded/blurry lens |
| | Macula | 103 | N/A |
| | Glaucoma | 97 | N/A |
| | Academy | 45 | School |
| | Vitamin (-s) | 189 | N/A |
| | Institute (s) | 41 | School; center |
| | Abnormal; Abnormality (-ties) | 51 | Rare; uncommon; unique; not normal; irregular; different |
| Cancer | | | |
| | Radiation | 1656 | X-rays |
| | Diagnose (-ed, -s) | 533 | Detect |
| | Chemotherapy | 718 | Chemo; drug |
| | Biopsy (-sied, -sies) | 919 | Sampling; remove; section |
| | Abnormal (-ly); Abnormality (-ties) | | Rare; uncommon; unique; not normal; irregular; different |
| | Microscope | 299 | N/A |
| | Carcinoma (-s) | 606 | Cancer type |
| | Immunotherapy | 300 | N/A |
| | Ultrasound (-s) | 180 | Sound waves |
| | Nausea | 101 | Motion sickness; seasick |
| Dementia | | | |
| | Alzheimer(-s) | 2655 | N/A |
| | Dementia(-s) | 1933 | N/A |
| | Caregiver(-s, -ing) | 698 | Carer |
| | Medication(s) | 327 | Drugs; capsule; tablet |
| | Diagnose(-s,-ed) | 195 | Detect |
| | Impairment | 106 | Other abled; damaged; hurt |
| | Abnormal (-ly); Abnormality (-ties) | 90 | Rare; uncommon; unique; not normal; irregular; different |
| | Vascular | 109 | Blood vessels |
| | Agitate (-ion, -ed) | 80 | Confused |
| | Hallucinate (-tion) | 58 | N/A |
| Cardiovascular Disease, Hypertension, and Stroke | | | |
| | Medication (-s) | 524 | Drugs; capsules; tablets |
| | Diabetes | 470 | High blood sugars |
| | Artery (-ies) | 578 | Blood vessel |
| | Cholesterol | 591 | N/A |

| | | | |
|---|---|---|---|
| | Cardiac | 281 | Heart |
| | Coronary | 305 | N/A |
| | Hypertension | 126 | High blood pressure |
| | Cardiovascular | 136 | Heart |
| | Rehabilitation | 142 | Rehab; healing |
| | Animation | 54 | Video images |
| Urinary Incontinence | | | |
| | Incontinence | 365 | No control |
| | Urine (-ate, -ation, -ary) | 360 | N/A |
| | Urethra (-al) | 188 | N/A |
| | Diary | 73 | Journal; daybook; blog |
| | Physician (-s) [Urologist (-s)] | 111 | Doctor |
| | Absorbent (-s) | 64 | Spongy |
| | Urgency | 43 | Hurry; haste; rush |
| | Abdomen | 27 | Stomach; belly |
| | Catheter | 78 | Tube |
| | Prescription (-s) | 27 | Drug |
| Kidney Associated Diseases | | | |
| | Dialysis | 904 | Kidney replacement |
| | Diabetes | 383 | High blood sugars |
| | Medication (-s) | 318 | Drugs; capsules; tabs |
| | Sodium | 148 | Salt |
| | Potassium | 174 | N/A |
| | Calcium | 220 | N/A |
| | Glomerular | 86 | N/A |
| | Phosphorous | 227 | N/A |
| | Urinary | 119 | N/A |
| | Filtering | 57 | Clean; process; clear |
| Osteoarthritis | | | |
| | Osteoarthritis | 247 | N/A |
| | Inflammation (Inflammatory) | 161 | Swelling |
| | Rheumatoid (Arthritis) | 480 | N/A |
| | Medication (-s) | 60 | Drugs; capsules; tablets |
| | Therapist | 31 | N/A |
| | Overweight | 32 | N/A |
| | Replacement (-s) | 42 | N/A |
| | Injection (-s) | 63 | Shot |
| | Rheumatologist | 19 | Joint and bone doctor |
| | Mobility | 26 | Movement |
| Osteoporosis | | | |
| | Osteoporosis | 821 | Soft and weak bones |
| | Calcium | 422 | N/A |
| | Vitamin | 283 | N/A |
| | Medication (-s) | 293 | Drugs; capsules; tabs |

| | | | |
|---|---|---|---|
| | (Pre-, Post-) Menopause (-al) | 98 | N/A |
| | Mineral (-s) | 86 | N/A |
| | (Rheumatoid) Arthritis | 96 | N/A |
| | Estrogen | 113 | Hormone |
| | Diagnose (-ed, -sis, -ing) | 58 | Detect |
| | Osteopenia | 38 | Low bone mass |
| Parkinson's Disease | | | |
| | Parkinson (-s) (PD) | 3183 | N/A |
| | Medication (-s) | 908 | Drugs; capsules; tabs |
| | Diagnose (-sis, -ed, -ing) | 357 | N/A |
| | Dopamine | 267 | N/A |
| | Levodopa | 271 | N/A |
| | Neurology [Neurologist (-s)] | 127 | Nervous system doctor |
| | Physician (-s) | 138 | Doctor |
| | Therapist | 138 | N/A |
| | Rigid (-ity) | 79 | Stiff |
| | Stimulation | 53 | Excite |
| Lung Associated Diseases | | | |
| | Oxygen | 1347 | |
| | Medication (-s) | 846 | |
| | Pulmonary | 845 | |
| | Diagnose (-ed, -s) | 441 | |
| | Inhaler (-s) | 337 | |
| | Fibrosis | 304 | |
| | Apnea | 253 | |
| | Hypertension | 223 | |
| | Nicotine | 177 | |
| | Rehabilitation | 170 | Rehab |

Note: Top 10 most frequent words by disease-cluster that were considered complex, either by syllabic count, character count, of unfamiliarity as described by the difficult word analysis. The frequency of the word in their respective disease-type patient education materials (PEMs) and potential alternatives are also depicted.

[a]Inclusion criteria for a "difficult word": 1) Any word with ≥3 syllables that was used at least once in ≥3 patient education material and; 2) was either unlisted on the New Dale Chal list of familiar words and the New General Service List;

[b]Alternatives selected are those that are considered synonymous while decreasing the individual word(s) syllable and/or character count.

**Supplementary Figure S1. Fry readability graph assessment of all high sentence estimate online patient education materials by disease cluster.**

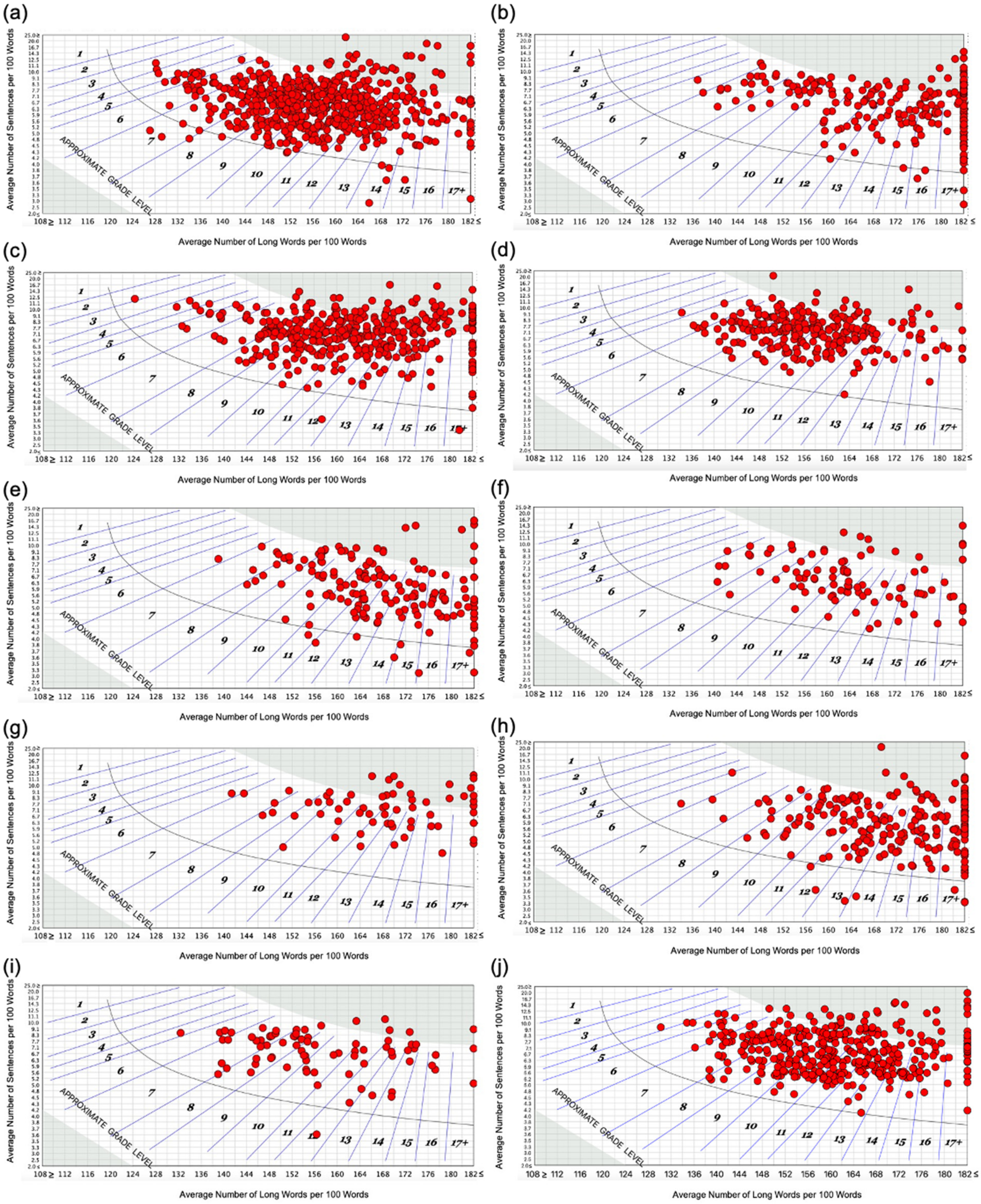


**Figure S1.** The Fry Readability Graph visually demonstrates the readability of articles by the intersection of the number of syllables per 100 words and the number of sentences per 100

words. Circles indicate reading levels of individual PEMs. The graph was generated using data from high sentence estimate patient education materials (PEMs) thereby displaying the lowest possible grade level each PEM would be able to achieve using the Fry Readability Graph. The graphs by disease types are as follows (a) Cancer, (b) Dementia, (c) Cardiovascular Disease, Hypertension, and Stroke, (d) Kidney Associated Diseases, (e) Age Related Macular Degeneration and Cataracts, (f) Osteoarthritis, (g) Osteoporosis, (h) Parkinson’s Disease, (i) Urinary Incontinence, and (j) Lung Associated Diseases.

**Supplementary Figure S2. Raygor readability estimate graph of all high sentence estimate online patient education materials by disease cluster.**

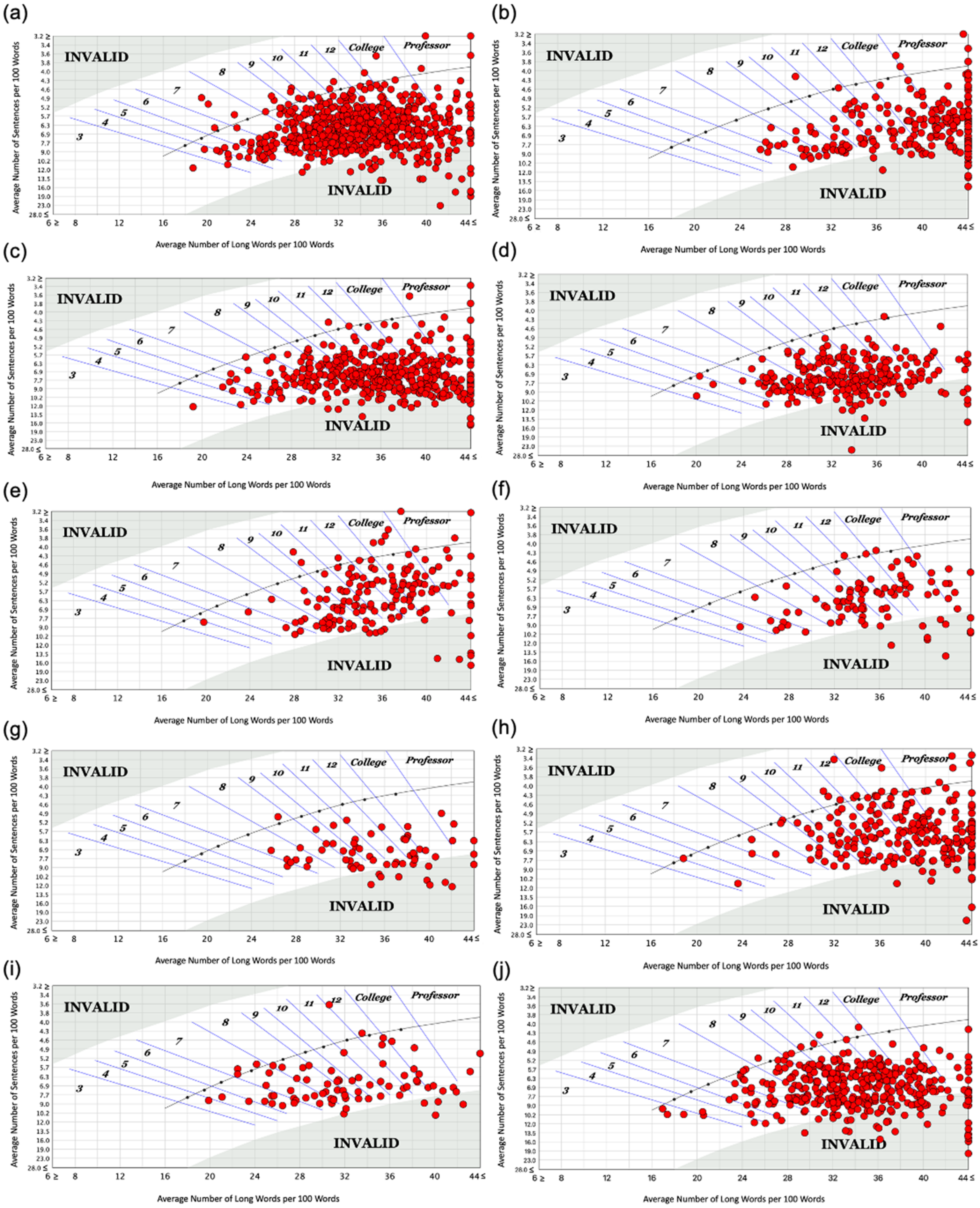


**Figure S2.** Raygor Readability Estimate Graph of all high sentence estimate online patient education materials (PEMs) collected from associations of chronic medical conditions. The

Raygor Readability Estimate Graph visually demonstrates the readability of the PEMs by the intersection of the amount of long words per 100 words and sentences per 100 words. Numbers within the graph indicate the approximate reading grade level. Circles indicate reading levels of individual PEMs. The graphs by disease types are as follows (a) Cancer, (b) Dementia, (c) Cardiovascular Disease, Hypertension, and Stroke, (d) Kidney Associated Diseases, (e) Age Related Macular Degeneration and Cataracts, (f) Osteoarthritis, (g) Osteoporosis, (h) Parkinson's Disease, (i) Urinary Incontinence, and (j) Lung Associated Disease.

**Supplementary Table S3.** Difficult words analysis displaying the mean and standard deviation of the % 3+ syllable words, % 6+ character words, and % unfamiliar words found in the patient education material (PEMs) of each of the disease clusters.

| Disease Cluster | % 3+ Syllable Words | % 6+ Character words | % Unfamiliar Word [a] |
|---|---|---|---|
| Age Related Macular Degeneration and Cataracts | | | |
| | 18.01 (+/– 4.75) | 35.79 (+/– 5.17) | 26.58 (+/– 6.08) |
| Cancer | | | |
| | 13.06 (+/– 4.65) | 33.87 (+/– 5.65) | 24.82 (+/– 6.18) |
| Dementia | | | |
| | 19.80 (+/– 5.41) | 39.60 (+/– 5.93) | 25.32 (+/– 7.58) |
| Cardiovascular Disease, Hypertension, and Stroke | | | |
| | 16.43 (+/– 5.30) | 36.12 (+/– 6.00) | 26.32 (+/– 6.85) |
| Urinary Incontinence | | | |
| | 13.53 (+/– 4.33) | 32.06 (+/– 5.61) | 24.33 (+/– 5.88) |
| Kidney Associated Disease | | | |
| | 12.73 (+/– 4.24) | 33.43 (+/– 4.66) | 23.10 (+/– 4.89) |
| Osteoarthritis | | | |
| | 16.97 (+/– 5.30) | 35.80 (+/– 5.49) | 27.08 (+/– 5.41) |
| Osteoporosis | | | |
| | 16.80 (+/– 4.94) | 35.42 (+/– 5.33) | 25.34 (+/– 5.62) |
| Parkinson's Disease | | | |
| | 20.47 (+/– 5.14) | 38.74 (+/– 5.72) | 27.38 (+/– 6.50) |
| Lung Associated Disease | | | |
| | 15.58 (+/– 4.26) | 34.04 (+/– 5.49) | 24.18 (+/– 6.22) |

[a] % Unfamiliar words as determined by the New Dale-Chall criteria. All analyses are reported as the mean and standard deviation (in brackets).

**Supplementary Table S4.** Difficult Word Analysis Statistics: Analysis of variance (ANOVA) and pairwise comparison of the difficult words analyses [e.g. the % 3+ syllable words, % 6+ character words, and % unfamiliar words found in the patient education material (PEMs) of each of the disease clusters].

| | **P Value** | **Pairwise Comparison of Parkinson's disease to other medical clusters' PEMs** [a] | | | | | | | | |
|---|---|---|---|---|---|---|---|---|---|---|
| **Difficulty Analysis** | **Across All PEMs** | **AMD & Cataracts** | **Lung Diseases** | **Cancer** | **Dementia** | **Heart & Stroke** | **Kidney Diseases** | **Osteoarthritis** | **Osteoporosis** | **Urinary Incontinence** |
| 3+ Syllables | <0.0001 | <0.0001 | <0.0001 | <0.0001 | ns | <0.0001 | <0.0001 | <0.0001 | <0.0001 | <0.0001 |
| 6+ Characters | <0.0001 | <0.0001 | <0.0001 | <0.0001 | ns | <0.0001 | <0.0001 | 0.0007 | 0.0008 | <0.0001 |
| Unfamiliar | <0.0001 | ns | <0.0001 | <0.0001 | 0.0112 | ns | <0.0001 | ns | ns | 0.0065 |

[a] p values for comparison across the different disease clusters' PEMs determined using analysis of variance (ANOVA). P values for pairwise comparisons between PEMs was determined using the Tukey's test for difficulty scores with significant difference ($P < .05$) across the forms on ANOVA. Note: ns indicated non-significance.
